# The Crossroads between Host Copper Metabolism and Influenza Infection

**DOI:** 10.3390/ijms22115498

**Published:** 2021-05-23

**Authors:** Ludmila V. Puchkova, Irina V. Kiseleva, Elena V. Polishchuk, Massimo Broggini, Ekaterina Yu. Ilyechova

**Affiliations:** 1 International Research Laboratory of Trace Elements Metabolism, ADTS Institute, RC AFMLCS, ITMO University, 197101 St. Petersburg, Russia; puchkovalv@yandex.ru; 2 Department of Virology, Institute of Experimental Medicine, 197376 St. Petersburg, Russia; irina.v.kiseleva@mail.ru; 3 Telethon Institute of Genetics and Medicine, Pozzuoli, 80078 Naples, Italy; epolish@tigem.it; 4 Istituto di Ricerche Farmacologiche “Mario Negri”, IRCCS, 20156 Milan, Italy; massimo.broggini@marionegri.it; 5 Department of Molecular Genetics, Institute of Experimental Medicine, 197376 St. Petersburg, Russia

**Keywords:** influenza virus replicative cycle, copper metabolism, ceruloplasmin, silver nanoparticles

## Abstract

Three main approaches are used to combat severe viral respiratory infections. The first is preemptive vaccination that blocks infection. Weakened or dead viral particles, as well as genetic constructs carrying viral proteins or information about them, are used as an antigen. However, the viral genome is very evolutionary labile and changes continuously. Second, chemical agents are used during infection and inhibit the function of a number of viral proteins. However, these drugs lose their effectiveness because the virus can rapidly acquire resistance to them. The third is the search for points in the host metabolism the effect on which would suppress the replication of the virus but would not have a significant effect on the metabolism of the host. Here, we consider the possibility of using the copper metabolic system as a target to reduce the severity of influenza infection. This is facilitated by the fact that, in mammals, copper status can be rapidly reduced by silver nanoparticles and restored after their cancellation.

## 1. Introduction

For the last 100 years, various aspects of human life have been negatively affected by viral respiratory infections caused by the influenza virus, the respiratory syncytial virus, coronaviruses, and rhinoviruses, etc. Influenza viruses are a cause of recurrent seasonal epidemics, as well as five catastrophic pandemic outbreaks, which have affected hundreds of millions of people and taken a great toll on global and local economies [1]. The primary countermeasure against influenza infection is vaccination, which is especially important in the case of pandemics [2]. However, in instances when vaccination is not possible (e.g., medical contraindications for vaccination or an already ongoing infection), the use of various classes of antiviral chemical agents becomes a primary strategy. These agents typically suppress the function of important viral proteins (e.g., neuraminidase or the M2 protein) [3]. As the viral genome is constantly changing, the virus quickly acquires drug resistance and can evade the immune response induced by vaccination. This genomic variability of the influenza virus significantly limits the efficiency of infection control [4]. Thus, the search for new tools to prevent and effectively treat acute viral respiratory infections is always of great interest.

One of the promising modern approaches to suppressing viral reproduction is the search for metabolic stages that are specifically changed by the virus. Many studies show that the activities of the infected cell are completely reprogrammed according to the viral strategy, with changes affecting the transcriptome, proteome, metabolome, and ionome. Recently, these studies have led to the proposal of the “virocell” concept [5,6,7,8]. According to this concept, rather than searching for pharmacological targets in the viral genome/proteins, they are located within the metabolic systems of the host cells themselves. Targeting these systems has an indirect influence on viral replication, thus potentially protecting the organism from influenza infections irrespective of virus and any acquired resistance to currently used antiviral agents.

Within this concept, an intensive search is conducted to determine metabolic stages that are affected by therapeutic agents; a central consideration here is that the target modifications should cause no significant harm to the host organism but should have destructive effects on viral replication [9]. It is important to consider that every stage of the viral life cycle relies on many host proteins, which affect the outcome of the infection, including the productivity of viral progeny, tropism, and pathogenicity. Concurrently, changes to cell metabolism are quantitatively assessed to evaluate their scale and any peculiarities in various cell lines and lineages, which are used in studies of viral infection. The accumulation of these data facilitates the creation of cellular model systems to evaluate therapeutic targets and approaches [10]. A new area of studying and developing agents that affect cell signaling pathways employed by influenza A virus (IAV) for its replication [11,12,13,14,15] also implicates copper-dependent pathways. An increasing amount of evidence demonstrates that IAV replication depends on cellular copper balance, and, possibly, is organism-wide [16,17,18]. It would be very important to identify the parts of the copper homeostasis system that may be targeted to counteract the influenza infection.

## 2. Influenza A Virus (IAV)

Data on various aspects of the lifecycle of the influenza virus (the morphology of the IAV virion, structure and function of viral proteins, infection factors and factors limiting IAV infection, implementation of the viral genome strategy, as well as the effect of the virus on host cells and the whole organism) are considered in modern reviews [19,20,21,22,23,24,25,26,27,28,29,30,31]. Here, the details of influenza virus biology, specifically the IAV replication cycle, are reviewed in relation to cellular copper metabolism.

Virus morphology, and viral proteins and genome. IAV, a member of the Orthomyxoviridae family, is an enveloped virus. Its virions may have a roughly ellipsoidal shape of a 100–120 nm diameter (typically for laboratory-adapted strains and for passaging virus in embryonated chicken eggs) or a filamentous morphology of about 300 nm diameter (characteristic of clinical isolates of IAV). The outer layer of the viral envelope is a membrane that is composed of a lipid bilayer from the host cell and three integral transmembrane viral proteins. The hydrophilic domains of two viral N-glycoproteins, hemagglutinin (HA) and neuraminidase (NA), are exposed at the surface of the membrane. HA coordinates host recognition and attachment and is also responsible for the fusion of viral and target cell membranes and for the release of the viral RNA genome into the cytosol. NA is required for the detachment of newly formed virions from the host cell. In electron microscopy images, NA and HA look similar to spikes or knobs on the virus surface. The third transmembrane protein of the viral envelope is an M2 tetramer, which possesses proton-selective ion channel activity involved in both viral entry and the formation of new viruses during viral assembly and budding. HA and NA exist in the envelope with an average ratio of 4:1, while the M2 protein is less abundant (one M2 molecule per dozens of HA molecules).

The inner layer of the viral envelope is a scaffold of matrix protein M1. It supports the lipid bilayer from inside, providing the virion 3D structure and mechanical rigidity, and encloses the virion core with its inner side. On the outer surface of the matrix, M1 protein forms contact with the C-terminal domains of HA and NA; on the inner surface, it interacts with virion core proteins.

The IAV core contains eight negative-sense single-stranded RNAs (vRNAs) segments, which comprise the viral genome. The 5′- and 3′-ends of each vRNA are self-complementary. Thus, a large RNA hairpin with a long stem is formed, which is further folded by intramolecular interactions into a compact vRNA secondary structure [24]. Each vRNA is associated with a set of proteins forming a ribonucleoprotein (vRNP). The 5′:3′termini of vRNA are bound by heterotrimeric RNA-dependent RNA polymerase complex, containing three subunits: PA, PB1, and PB2 (the codes originate from chemical properties of the proteins: acidic, basic 1, and basic 2, respectively). Copies of arginine-rich nucleoprotein (NP) are associated with the double-strand region of the vRNA stem. Both NP and the RNA-dependent RNA polymerase complex contain nuclear localization signals as well as nuclear export signals. In addition to vRNPs, the core contains the nuclear export protein (NEP; also called nonstructural protein 2, NS2) [32,33]. vRNAs have highly conserved noncoding regions at both the 3′ and 5′ ends, which are used as cis-elements for regulation of replication and transcription by the viral polymerase complex, as they also take part in packaging during virus assembly. The virion also contains an NS1 protein, which was initially referred to as nonstructural (NS1, nonstructural protein 1). This protein possesses many functions, allowing interactions between the virus and the host, aiding viral replication and suppressing the innate immune system. [34,35].

In addition to viral proteins, the virion typically contains several host proteins in minor quantities (CD9, CD81, annexins, cytoskeleton components, etc.). The role of specific host proteins in the IAV life cycle is disputed; the estimates range from negligible to very important. The virus contains 40–50 unanchored polyubiquitin chains with free C-termini, which are recruited from the host cell during virion assembly and used for the release of vRNPs in the next infection cycle [36,37].

**Figure 1:**
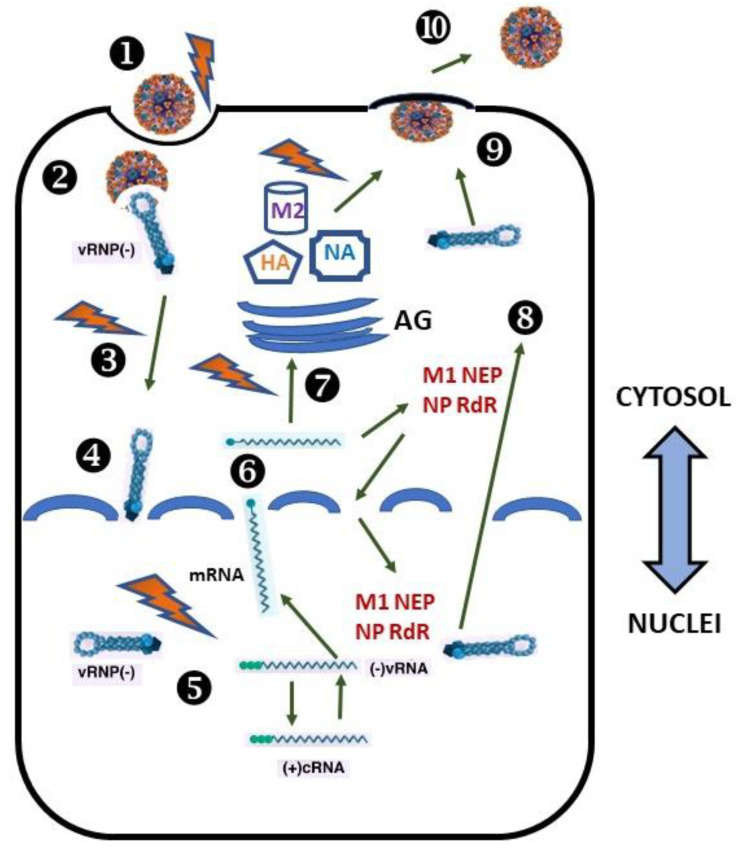
Schematic representation of the important steps of the IAV life cycle. *Recognition and attachment (Stage 1).* Viral HA (orange) recognizes the terminal α2,6- or α2,3-linked NANA residues in glycan of the sialo-containing receptor on the plasma membrane and attaches to them. *Entry (Stage 2).* Virus entry to the host cell via endocytosis. *Fusion of endosomal and viral membranes and release of viral genome (Stage 3).* Once inside the acidic endosomal compartment and after the structural rearrangement of HA, the lipid bilayers of the virus and endosome fuse, and a pore is formed. *Transfer to nuclei (Stage 4).* Viral genome packed in vRNPs is released into the cytosol through the formed pore. *Transcription, replication, and assembly (Stage 5).* Later, they are transferred to the nuclei. Transcription and replication of the IAV genome occur at the genomic vRNA in the nuclei. *Synthesis and maturation viral proteins (Stage 6 and 7)*. v-mRNAs are transported into the cytosol and translated on membrane-bound or free polyribosomes. *Packaging of vRNPs (Stage 8).* Viral proteins that are needed in virus assembly (M1, NP, NEP, and RNA-dependent RNA-polymerase subunits) return to the nucleus to be packaged as vRNPs. Later, they are exported to the cytosol and delivered to the plasma membrane. *Budding and realizing virus particles (Stage 9 and 10).* Envelope viral proteins (HA, NA, and M2) are synthesized on the rough ER and enter the cellular secretory pathway. Here, HA and NA co-translationally acquire the N-carbohydrate block, which completes their maturation in the Golgi. After that, they are transported via post-Golgi transport machinery to the PM and meet the vRNPs. Here, the budding and release of progeny viruses occur. Protein M1 assists in the formation of mature virions, using fragments of the lipid bilayer and some proteins of the host cell. The viral NA cleaves sialic acid from cell-surface glycoproteins and glycolipids, and virions are released.

IAV reproduction. The life cycle of IAV consists of different stages: (i) cell entry, (ii) RNA and protein production, and (iii) virion assembly and exit from the host cell. The main cellular compartments of the host cell implicated in viral propagation are the endosomal compartment, cytosol, nucleus, endoplasmic reticulum (ER), and the Golgi complex (Figure 1).

(i). IAV cell entry. IAV cell entry starts with recognition of a potential host and the attachment of virion to the cell membrane. On the host cell surface, viral HA recognizes terminal residues of N-acetylneuraminic (sialic) acid (NANA), a carbohydrate moiety of N-glycoproteins, belonging to receptors of the endocytic pathway. HA recognizes NANA residues, linked to galactose through carbon-3 or carbon-6 (α-2,6- or α-2,3-linkages). In humans, α-2,6-linkages predominate in tracheal epithelial cells while α-2,3-linkages are more common in the respiratory epithelium [38]. Attachment of the virion to the receptor initiates clathrin-mediated endocytosis, and the virus is captured into the endosomal compartment. Other IAV cellular entry pathways have also been described, including caveolae-mediated endocytosis and micropinocytosis; however, the details of these processes are less clear [39]. In endocytic vesicles containing the virus, the concentration of protons inside this acidic compartment is increased through the action of vATPase. Then, due to the activity of M2 protein, a proton-selective ion channel, protons actively enter the viral core. The acidity continues to increase, leading to disruption of the protein–protein interaction inside the viral particle. Consequently, matrix proteins detach from the proteins covering the RNA genome and the process of uncoating starts.

In addition, low pH (acidity) induces a conformational change in the HA molecule, promoting its cleavage by a trypsin-like host protease into two subunits: HA1 and HA2. HA2 subunits undergo a transition leading to a coiled-coil structure of three α-helices that play critical roles in the targeting and direct fusion of the viral particle membrane with the endosomal membrane and that play critical roles in targeting and direct fusion of the viral particle membrane with the endosomal membrane and in a pore formation. Moreover, conformational change in the M1 protein under acidic pH leads to the disruption of M1 protein contacts with HA, vRNPs, as well as its intermolecular contacts. All of these events lead to a release of vRNPs to the cytosol through the pore produced by the fusion of viral particles with the endosomal membrane. [40]. Unanchored polyubiquitin chains play an important role in the release of vRNPs; they are recognized by cytosolic histone deacetylase 6, a key factor for the assembly of microtubule-dependent or microfilament motor complexes. These complexes provide the force, which facilitates the release of vRNPs to the cytosol [41].

(ii). RNA and protein production. After the uncoating process, vRNPs are transported to the nucleus. This is mediated by the small GTPase karyopherin β2 and the presence of nuclear localization signals in RNA polymerase and the NP protein. Karyopherin β2 binding to vRNPs induces their complete dissociation from M1 proteins. In the nucleus, viral RNAs are processed by the viral RNA-dependent RNA polymerase. Negative-sense vRNA is used as a template for the synthesis of two types of positive-sense RNA: for transcription and for replication [42]. IAV uses the alternative splicing mechanism as a part of the viral genome strategy to enhance the information capacity, so several different v-mRNAs can be formed from one vRNA segment. Transcribed mRNAs contain the cap and poly(A) tract, which are required for export to the cytosol and recognition by eukaryotic translation factors. Caps are obtained by the “cap snatching” mechanism (the PB1 and PB2 proteins steal short 5′-capped transcripts produced by the cellular RNA-polymerase II, which are then used to prime transcription of viral mRNA) [43]. The poly(A) tract of viral mRNAs is encoded by genomic vRNAs as a tract of 5–7 uracil residues [44,45].

Mature v-mRNAs associate with the cap recognition complex, poly(A) binding protein, and other nuclear proteins that facilitate mRNA transport, thus using the host mRNA export system for their delivery to the cytosol. Here, viral mRNAs are translated by the host ribosomes. The virus uses several strategies to increase the proteome capacity by alternative translation of v-mRNAs: the presence of several alternative start codons, frameshifts, alternative open reading frames, etc. V-mRNAs that encode three proteins of the outer layer of the viral envelope (HA, NA, and M2) also encode N-terminal signal peptides, which ensure the binding of these nascent peptides to the membranes of the rough endoplasmic reticulum and their entry into the lumen of the secretory pathway. Here, HA and NA extracellular domains are co-translationally N-glycosylated and, then, the proteins are transferred to the Golgi, where the maturation of the carbohydrate moieties is completed, and finally transferred to the plasma membrane. The other viral proteins are synthesized in the cytosol. The soluble cytosol proteins contain signals that direct them to the nucleus to virion assembly. Each replicated genomic negative-sense vRNA (a perfect copy of the negative-sense vRNA of the initial virus) [42] adopts a hairpin structure and binds the RNA polymerase complex at its 3′-5′ double-strand stem, while NP proteins are associated with the RNA throughout its length. Thus, vRNA, RNA-polymerase, and NPs form eight vRNPs.

(iii). Virion assembly and exit from the host cell. GTP-dependent importins are responsible for the delivery of vRNPs to the cytosol through the nuclear pore complex. vRNPs that entered the cytosol bind to recycling endosomes (RE). There, every RNP binds cytosolic M1, and the vRNPs start to associate with each other. vRNAs have specific packaging signals in the 3′- and 5′-regions and along the double-stranded region, which can bind adjacent vRNAs through Watson–Crick pairing (kissing loop complex), forming the genomic core of the virion [24,46]. The sequence of assembly of the vRNPs complexes may vary. The most experimentally supported schematic of IAV assembly is (7 + 1): seven vRNPs assemble laterally with their 3′ and 5′-ends arranged in similar orientations with the eighth vRNP in the middle [47]. Transport vesicles, which bud from the Golgi, carry HA, NA, and M2 proteins to the apical domain of the cell membrane. Their fusion with the membrane occurs in the raft-like regions enriched in sphingolipids and cholesterol [48]. The binding event between the M1 protein and the C-terminal domains of HA, NA, and M2 stimulates clustering and assembly of the IAV protein envelope and marks the beginning of virus budding at the plasma membrane. Packed and assembled RNA proteins of the viral genome are delivered to the budding site of the plasma membrane by microtubule-dependent transport. The accumulation of HA and NA in lipid rafts stimulates bud growth and closure, leading to the release of virus progeny from the cell, eventually bringing about infection of other cells. The viral NA cleaving sialic acid from the cell-surface glycoproteins and glycolipids actively participates in this process.

## 3. The Biological Roles of Copper and Its Turnover in the Mammalian Body

Copper is an essential micronutrient for almost all modern organisms. In mammals, it is the third most abundant trace element after iron and zinc [49]. Two biological functions of copper are well documented. First, it is a catalytic and structural cofactor of vitally important enzymes, which play a key role in cellular respiration (cytochrome-c-oxidase, CCO), the synthesis and degradation of biological amines (dopamine-beta-hydroxylase, tyrosinase, and monoaminoxidase), the activation of neuropeptides (peptidylglycine alpha hydroxylating monooxygenase). Moreover, copper neutralizes toxic ROS (cytosolic and extracellular Cu(II)/Zn(II)-superoxide dismutases, SOD1, and SOD3), takes part in bidirectional iron transport through the cell membrane (ceruloplasmin, Cp, hephaestin, and zyklopen), and provides the formation of connective tissue (lysyl oxidase), etc., [50,51]. The most abundant cuproenzymes catalyze electron transfer to dioxygen, owing to the ability of copper to change its oxidation state Cu(I)/Cu(II) in the biologically relevant redox potential and pH ranges [52].

Second, copper is a fundamental element of several signaling pathways and a co-activator of key transcription factors. For example, copper is required for the following systems: X-linked inhibitor of apoptosis protein pathway [53], MAPK (Ras/mitogen-activated protein kinase) pathway [54], and BRAF (Raf of murine oncogene sarcoma homologue B1-dependent) pathway [55]. Copper modulates the function of growth factor receptors [56], gamma-aminobutyric acid type A (GABA(A)) receptors, N-methyl-D-aspartate (NMDA) receptors, and voltage-gated Ca(2+) channels [57] and regulates cyclic-AMP-dependent lipolysis [58]. Furthermore, intracellular and local extracellular changes in the copper concentration influence the activity of nuclear factor kappa-light-chain-enhancer of activated B cells (NF-kB) [59] and hypoxia-inducible factor 1 (HIF1α) [60]. These factors regulate the expression of several dozens of genes, including genes for which the products take part in the reprogramming of energy metabolism in tumor cells. Moreover, copper is required for the induction of Golgi-complex-independent secretion of interleukins and cytokines [61] as well as for odorant receptor activation [62]. It has also been shown that copper controls organogenesis and cell differentiation in embryo development [63].

**Figure 2:**
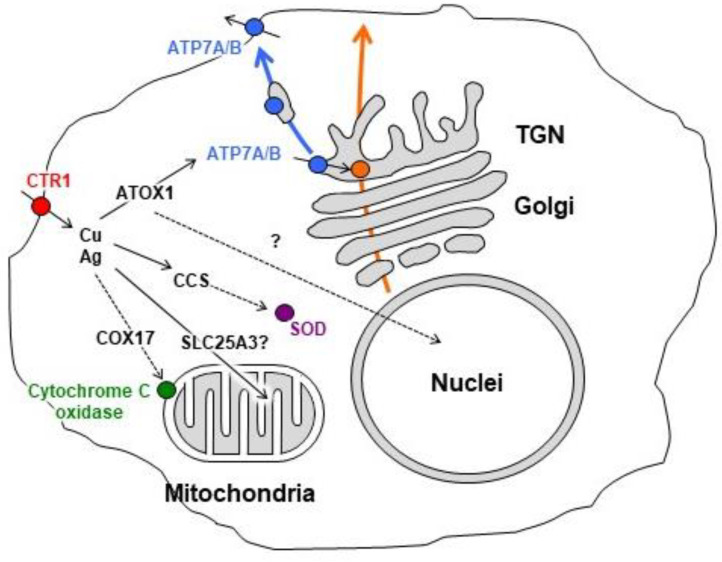
Scheme of copper distribution in a mammalian cell. Bulk copper is taken up via CTR1. After being imported into the cell, the copper is transferred to copper chaperones for SOD1 (CCS), copper chaperones for cytochrome-c-oxidase (COX17), and copper chaperones for copper-transporting ATPases (ATP7A/B) antioxidant protein 1 (ATOX1). In turn, ATP7A/B transmits copper into the Golgi lumen. In the Golgi, ATP7A/B loads copper on newly synthesized cuproenzymes, which transport it along the biosynthetic pathway. In the cells, the Cu(I)/Cu(II) redox cycle including metallothionein Cu(I), glutathione Cu(I)/Cu(II), and COMMD1 (CuII) exists.

Vital functions of copper are invariably accompanied by high toxicity, as uncoordinated copper ions catalyze the formation of uncontrolled reactive oxygen species (ROS) [64] and cause oxidative stress, which promotes the development of neurodegenerative, oncological, metabolic, and cardiovascular diseases [65]. For this reason, mammalian organisms possess precisely controlled systems, which safely transport Cu from the gastrointestinal tract to sites of cuproenzyme formation [66] (Figure 2).

The normal daily intake of dietary copper by adult humans is about 2 mg. In the acidic environment of the stomach followed by the breach of copper sites in proteins, copper ions are released from food and coordinated by amino acids (most probably by histidine or cysteine) and small peptides. In this way, copper is preserved in a soluble and biologically safe form, in which it is transported to the small intestine, where it is absorbed [67]. The amount of intestinal copper absorption is strongly regulated by body copper concentration. Dietary copper is transported to enterocytes as ions through DMT1 (Divalent Metal (Ion) Transporter 1) and universal copper transporter CTR1 (high-affinity copper uptake protein 1) channels, which are resident proteins of the enterocyte apical membrane [68,69]. Inside the cell, copper is bound by cytosolic chaperone ATOX1 (Antioxidant 1 Copper Chaperone) and transferred to metal-binding sites of ATP7A (ATPase Copper Transporting Alpha polypeptide, also known as Menkes disease protein), which actively pumps copper to the extracellular space of the body and into the bloodstream. There, copper is bound by serum albumin or α-2-macroglobulin, which carries it via the portal vein to the hepatocyte cell membrane [70]. Hepatocytes import copper through CTR1. Inside the hepatocytes, copper is distributed across several compartments. First, copper is transported from CTR1 directly to copper chaperones, which terminally deliver it to forming cuproenzymes of cytosol SOD1 and mitochondria CCO. This part of copper comprises the intracellular catalytic compartment. A fraction of copper is delivered into the lumen of the Golgi complex by ATP7B (ATPase Copper Transporting Beta polypeptide, also known as Wilson disease protein), where it metallates apo-ceruloplasmin (apo-Cp).

Holo-Cp is secreted into the bloodstream and comprises the major extracellular copper pool. Cp is a copper donor for non-hepatocyte cells. In addition, copper is bound by the system that includes metallothionein-Cu(I), glutathione (Cu(I)/Cu(II)), and COMMD1-Cu(II) (Copper Metabolism gene MURR Domain 1) [71,72,73]. Copper retained in this system can change its oxidation state. The system may share copper with Cu chaperones for cuproenzyme metalation, may provide copper for copper-required signaling factors, transcription factors (HIF1α; NF-κB, Sp1, etc.), and may replenish the mitochondrial pool of non-catalytic copper. The amount of copper that is not used by any of the listed compartments (excess absorbed copper) is excreted from the liver in the bile by the ATP7B protein, which is localized on the canalicular cell membrane of hepatocytes.

## 4. The Points in Which IAV-Induced Inflammation Cross with Copper Metabolism

*Point 1. SOD1 metalation*. Successful and effective replication of IAV requires an increase in cellular ROS production. It is well documented that deficiencies in ROS-generating enzymes, for instance, by gene knockout or selective inhibitors, reduce lung injuries after IAV infection [74,75,76,77,78,79,80]. Both the induction of ROS production and suppression of the host antioxidant system are used to boost ROS concentration in the IAV replicative cycle. In cells, ROS continuously arises from one-electron reduction of dioxygen to superoxide anion-radical, as a byproduct of electron transfer (electron leak). Superoxide anions, which are not very reactive per se, convert to hydrogen peroxide, hydroxyl radicals, and a variety of their reaction products such as organic hydroperoxides and hypochlorous acid [81]. Electrons causing ROS formation can leak from the respiratory electron chain of oxidative phosphorylation, reactions catalyzed by cellular NADPH oxidases, the NADPH oxidases/dual oxidases, and xanthine oxidase. In normally functioning cells, the constitutive production of ROS is thoroughly controlled, and ROS species are used as signal molecules that can modulate growth, synaptic transduction mechanisms, and other processes [82].

The main ROS-scavenging systems include the cytosolic and mitochondrial SODs, the catalases (in peroxisomes and in cytosol [83]), the peroxiredoxins, the glutathione peroxidases, the thioredoxins, and the glutathione pool; the balance between reduced and oxidized state of the latter is sophisticatedly regulated. The activity of genes responsible for antioxidant defense is regulated by transcription factors that contain thiol groups, which act as redox-sensitive switches. In subtoxic ROS concentrations, they increase the activity of genes that code antioxidant and ROS-scavenging proteins. This allows for the maintenance of the subtle control exerted on the cellular redox balance [84]. In cells infected with IAV, the activity of ROS-producing agents is stimulated [78,85]. The targets of IAV-specific suppression of the host cell antioxidant defense include all of its major members. The first among them is Cu/Zn-SOD1, a major cuproenzyme of the cytosol [86]. The functional SOD1 molecule is a homodimer, copper ion is its catalytic cofactor, while a zinc ion is a structural cofactor. SOD1 catalyzes dismutation (disproportionation) of the superoxide (O^−2^) anion radical into molecular oxygen (O_2_) and hydrogen peroxide (H_2_O_2_). It has the largest kcat/KM of any known enzyme (≈7 × 10^9^ M^−1^s^−1^) and retains its activity in a wide range of pH and temperature values [87]. The level of active holo-SOD1 is backed up by the presence of a permanent apo-SOD1 pool, which can be converted to holo-SOD by transferring a copper ion from the Cu(I)-chaperone to SOD1 (CCS) that obtains copper directly from CTR1 at the cell membrane [88,89]. Thus, damping the activity of SOD1 is not an easy task. Some viruses belonging to the Poxvirus family, which rely on the increase in ROS production, encode viral SOD-like proteins with no dismutase activity but with a high affinity to copper atoms. Such proteins recognize holo-CCS proteins and snatch copper ions from them. Viruses from the Chordopoxvirus subfamily encode apo-SOD-like proteins that do not have copper-binding sites. Such proteins efficiently bind holo-CCS and competitively suppress copper loading to host apo-SOD. Both types of proteins reduce the activity of SOD1; the expression of these proteins increases virus production, while the loss of the respective genes is detrimental for the virus [90,91]. However, mammalian apo-SOD1, due to the presence of dual prolines near the C terminus of the polypeptide, may obtain copper in CCS-independent ways, possibly with the help of the reduced form of glutathione [92,93]. Thus, the anti-SOD strategy of these viruses seems to be imperfect.

Suppression of SOD1 activity by IAV could be different. It has been demonstrated that, in IAV-infected A549 cells (originating from adenocarcinomic human alveolar basal epithelial cells), SOD1 gene activity drops to practically zero both at the transcription and translation levels, while the content of virus-specific proteins grows proportionally with infection dose. Both the N-acetyl-cysteine (abiogenic antioxidant) and SOD1 overexpression neutralized IAV-induced ROS production and enhanced cell survival [80,94]. IAV-induced inhibition of SOD1 expression is mediated by the suppression of Sp1 (specificity protein 1) gene activity. IAV infection causes a dramatic decrease in the level of mature Sp1 gene transcripts. Concurrently, existing Sp1 molecules are subject to cleavage and degradation in a proteasome-mediated pathway [80]. The drop in Sp1 protein levels causes a decrease in SOD1 gene expression as the proximal part of the SOD1 gene promoter contains a cluster of *cis*-elements (GC-rich box) for the Sp1 protein, which is a constitutive transcription activator of housekeeping genes as well as genes with TATA-less promoters [45,95,96]. The existing data allow for speculation that the SOD1 metalation pathway may be viewed as a copper-dependent element of IAV replication and a potential target of antiviral strategies.

*Point 2. ATP7A/autophagosome pair*. The productive replication of IAV induces autophagy in the host cell; this cellular process represents one of the ways by which long-lived proteins and damaged organelles are degraded [97,98]. IAV-induced autophagosome biogenesis is used by the virus at different stages of replication: for the release of the viral genome from endosomes as well as the production of infectious viral particles [98]. Moreover, autophagy also leads to a decrease in SOD1 activity [99]. The relation between ATP7A and autophagosome activity has been established, and the importance of both pathways for effective copper export has been shown [98]. Inhibition of autophagosome formation by spautin (a specific synthetic inhibitor of autophagy) deranges dengue virion maturation [100], possibly by blocking the autophagosome-mediated pathway of copper export [101,102]. Thus, the ATP7A/autophagosome pair may be viewed as an element of copper metabolism that may be targeted to block IAV reproduction.

*Point 3. Copper import and excretion*. The first convincing evidence that efficient IAV replication requires a normal physiological balance between copper import and copper excretion was obtained in A549 cells with RNAi knockdown of the CTR1 or ATP7A genes [103]. These copper transporters with opposite functions in intracellular copper balance were both required for IAV replication. CTR1 or ATP7A deficiency resulted in the corresponding decrease or increase in cellular copper concentration. In both cases, the virus RNA concentration, and the levels of specific virus proteins, as well as extracellular virus titer, decreased. However, a rough abiogenic increase (CuCl_2_ was added in the culture medium) or decrease (copper was captured by tetrathiomolybdate) in intracellular copper concentration did not significantly affect virion assembly, morphology, or the release of viral particles. These observations indirectly indicate that copper-dependent stages of IAV replication occur in cell compartments that rely on CTR1 and ATP7A for copper import and excretion. Therefore, CTR1 and ATP7A might be considered elements of copper metabolism critical for the vital function of IAV.

*Point 4. Prion protein*. In recent years, evidence for the influence of the native prion protein (PrPC) on IAV replication has been increasing. The prion proteins are mostly expressed in brain cells but have also been found in the heart, skeletal muscles, intestinal tissues, uterus, testes, and cells of the immune system [104]. The prion protein is notorious for its ability to conformationally convert from its native soluble and proteinase-sensitive isoform PrPC to the PrPsC isoform that aggregates in insoluble, proteinase K-insensitive fibrils, which are responsible for deadly “conformational” diseases [105,106]. The role of PrPC in the pathogenesis of prion conformational diseases is universally recognized, but its normal physiological role is still unclear. The phenotypes of PrP-null mice have been uninformative: they did not demonstrate any major anatomical or developmental deficits [107]. Moreover, the mice in which the PrP gene was deleted postnatally were phenotypically relatively normal [108].

It is widely accepted now that PrPC takes part in copper metabolism. This assumption is in good agreement with the data on the structure and biochemical properties of PrPC. PrPC is bound to the trans-lipid layer of the cell membrane through the glycosyl phosphatidylinositol anchor (GPI-anchor) [109,110]. The protein consists of two short beta-strands and three stabilizing alpha-helices. The N-terminal domain of PrPC, comprising about 100 amino acid residues, is natively unstructured. In this domain, a conservative sequence motif is localized, which is formed by nonapeptide PQGGGGWGQ, followed by four similar octapeptide repeats (PHGGGWGQ). Each octapeptide repeat can bind a single Cu(II) ion. Two residues, His96 and His111, can bind two more Cu(II) ions. Thus, the N-terminal domain of PrPC can bind up to six copper(II) ions. The *Ka* for copper binding to PrPC falls into the femtomolar range (10^−14^ M), so PrPC binds copper within the physiological concentration range [111]. At the same time, the binding of other divalent cations such as Ni(II), Zn(II), or Mn(II) to PrPC is weaker by three or more orders of magnitude [112]. These properties allow PrPC, as a member of the copper homeostatic system, to operate as an agent for Cu(II) transport into the cell. One of the proposed copper-dependent functions of PrPC is the suppression of ROS toxicity, possibly through modulation of the SOD1 function [110]. As CTR1 has significantly less affinity for copper (Km ≈ 2·10^−6^ M) [113] than PrPC, it is quite improbable that PrPC cannot pass copper to CTR1 on the cell membrane. Prion-mediated copper transport and its connection to intracellular copper pathways should use alternative routes. The mechanism of PrPC-related copper transport is possibly linked to caveolin 1-, clathrin-, and raft-dependent endocytosis [104]. In any case, copper-carrying PrPC ends up in the lysosomes. At low pH values, copper ions dissociate and can enter the cytosol via STEAPs [114].

The work of Japanese research groups has persuasively shown that PrPC has a protective role in influenza A infection. To prove this concept, they demonstrated that the prion protein is actively expressed in the lungs. PrPC is found in lung epithelial cells, including alveolar and bronchiolar cells. Lung tissue has the largest quantities of PrPC among nonneuronal tissues, and the gap between the concentration of PrPC in the brain (which contains the major fraction of this protein) and in the lungs is not so large [115]. In mice lacking PrPC (Prnp0/0), IAV infection caused greater mortality, more extensive lung damage, higher lung virus titer, and larger weight loss compared to wild-type mice. In the lung tissue of Prnp0/0 mice, ROS concentration and xanthine oxidase activity were elevated while SOD1 activity decreased. The ectopic expression of the PrP gene without the copper-binding domains in Prnp0/0 mice did not restore the normal level of resistance to IAV infection. These combined data allowed the authors to suggest that PrPC protective function is linked to its copper-binding activity and rescue of SOD1 [115,116]. It is possible that SOD1 is metalized via a CCS-independent way [93].

Moreover, the data obtained by the same group of researchers revealed another possible mechanism of PrPC-mediated protection of mice from IAV infection. It is linked to the ability of PrPC to induce anti-inflammatory M2 macrophage polarization via activation of Src family kinases (SFKs). Activated M2 macrophages suppressed inflammation and protected mice from the lethal effect of IAVs, possibly by preventing the development of a cytokine storm [117].

*Point 5. Ceruloplasmin (Cp)*. The initial data showing the relationship between Cp gene expression and IAV infection were obtained more than 50 years ago [118,119]. Afterwards, these observations were supported by many investigations demonstrating that the level of Cp-associated oxidase activity of blood serum increases several times during influenza infection. The increase in serum holo-Cp concentration begins during the early stage of the infection with a high Cp level persisting throughout the infection period and positively correlating with disease severity [120,121]. The effect is possibly caused by an elevation of cytokine levels characteristic of IAV infection. The Cp gene promoter contains *cis*-elements for interleukins; thus, the Cp gene expression is upregulated during infection [122,123]. However, the specific role of Cp in the IAV replicative cycle is not firmly established at present.

Cp is a major copper-containing N-glycoprotein of the α-2-globulin fraction of vertebrate blood serum. It is coded by a unique gene containing 20 exons; the gene structure is very similar to vertebrates from fish and mammals [124,125,126]. Two isoforms of Cp exist: the first is an extracellular soluble form (sCp), and the second is a membrane-bound form (GPI-Cp), which is anchored to the plasma membrane by a glycosil phosphatidyinositol (GPI) anchor. The isoforms are coded by two molecular variants of Cp-mRNA, which are formed by alternative splicing of Cp gene transcript. sCp is synthesized by the cells of organs that produce extracellular fluids: the liver [127], mammary gland [128], and choroid plexus [129,130]. GPI-Cp is synthesized in Sertoli cells [131], glial cells of various brain regions [129,132,133], peripheral blood lymphocytes and macrophages, and hepatocytes [134].

A functional Cp protein is formed by a single N-glycosylated polypeptide chain and 6–8 copper ions; Cp primary and tertiary structures are similar in distant evolutionary groups of vertebrates (fishes, amphibians, reptiles, birds, and mammals). In all of the studied species, the Cp molecule has a multidomain structure and consists of slightly more than 1000 amino acid residues and 2–3 carbohydrate blocks with the N-acetylneuraminic acid (NANA) in a terminal position. In most cases, NANA is bound to a galactose residue through a carbon-6 by α-2,6-bond; the same type of bond is recognized by influenza HA [135,136]. Three active centers contain six copper atoms, which are often classified based on their spectral properties (two type 1 and one type 2 centers, and one trinuclear center). Cp molecules can also contain up to two loosely bound non-catalytic copper atoms. Cp belongs to the family of multicopper blue (ferr)oxidases; it is a multifunctional moonlighting protein with enzymatic and nonenzymatic functions [137,138].

Cp has many functions, two of them are universally accepted. The ability of Cp to catalyze the Fe(II) → Fe(III) transition and to provide bidirectional iron transport across the cell membrane is considered its main biological activity [139,140,141]. The importance of GPI-Cp (in its complex with ferroportin) for iron release from cells is well documented [142]. The critical role of Cp in iron homeostasis in vivo is supported by the clinical picture and the biochemical manifestation of aceruloplasminemia (a rare autosomal recessive disorder caused by mutations in the coding region of the Cp gene that lead to a deficiency of expressed Cp protein). Similar disorders are observed in mice with a targeted Cp gene deletion [143,144]. The second function of Cp is non-catalytic: it can transport copper to the cells of non-hepatocyte organs. This function was convincingly proved in recent studies [145]. Besides these two functions, which were proven in vivo, Cp can oxidize biogenic aromatic amines (adrenaline, noradrenaline, serotonin, and dopamine) as well as abiogenic diamines [146] from dioxygen; it belongs to the group of acute-phase proteins [147], displays weak superoxide dismutase activity [148] and glutathione-linked peroxidase activity [149], and can control neutrophil apoptosis [150]. Only native holo-Cp can perform the mentioned functions.

In humans, more than 95% of blood serum copper is associated with Cp [151]. The main fraction of blood Cp is synthesized by the liver parenchymal cells [127]; small amounts of Cp are secreted by activated macrophages and lymphocytes [152,153]. In the liver, which plays a central role in the copper balance of mammalian organisms, Cp peptide chains are synthesized by membrane-bound polyribosomes. They are N-glycosylated co-translationally in the lumen of rough ER. In the lumen of the Golgi complex, copper atoms are added by copper-transporting P1-type ATPase (ATP7B, Wilson ATPase) [154]. Carbohydrate moieties also mature and obtain a unique structure in the Golgi. Copper is an essential structural and enzymatic cofactor of Cp; indeed, folding to the native state requires proper loading of copper atoms [155].

As mentioned, the level of Cp in a patient’s blood serum rises several times and remains high during IAV infection [120,121,156]. This effect was viewed as a defensive response of the organism, and Cp was considered a potential nonspecific agent for prophylaxis and treatment of myxo- and paramyxovirus infections [118].

The researchers from the Institute of Virology (Bucharest, Romania) proposed a “trapping” hypothesis, in which Cp suppresses the IAV infection by trapping the virus in complexes of viral envelope glycoproteins with Cp carbohydrate moieties [135,157]. This hypothesis was supported by the following experiments: the major glycoproteins of the virus envelope’s HA and NA membrane that determine the infectivity interact with Cp and asialo-Cp and form complexes with various degrees of stability. Virions, in which the HA and NA were blocked by Cp, were no longer able to interact with the plasma membrane glycoproteins of host cells and to infect these cells. Kinetic constants for IAV binding to Cp corresponded well with substrate-specific interaction.

The “trapping” hypothesis was confirmed by the data on the relationship between blood Cp concentration and IAV replication. The inhibitory effect of Cp was observed for virus replication in chicken embryos, chorioallantoic membrane fragments, and when administered to mice. The effect depended on the method of administration [158]. Thus, the strongest inhibition of the virus replication was observed when the virus was incubated with Cp prior to inoculation. The administration of Cp simultaneously with the virus or after inoculation was not effective either in vitro or in vivo. The largest protective effect was obtained when mice were injected with Cp prior to IAV inoculation [159,160]. The effect had a pronounced dose dependency. Surviving mice, which received Cp prior to inoculation, displayed the highest levels of protection against reinfection [161]. The authors of the “trapping” hypothesis proposed that Cp acted as a trap for virions at early stages of infection, while at late stages of infection, Cp acted as an extracellular superoxide dismutase and neutralized products of the oxidation stress caused by the virus [157].

The experimental approaches and methods used by the authors of the hypothesis give no reason to doubt the quality or reliability of their results. However, the existing results did not answer several questions. A central remaining question is whether the presence of Cp in the extracellular space of the respiratory tract is at concentrations that would be sufficient for the formation of complexes or aggregates with virus particles and could block the infection of lung cells. Simply put, is Cp synthesized in the lungs in necessary amounts? It was shown that, in rat lungs, only one transcription product of the Cp gene is formed; its level increases during embryonic development and then drops to undetectable levels in the postnatal period [162]. At the same time, inflammation induced by endotoxins in the lungs of adult rats was characterized by a significant increase in Cp gene activity [163]. These data were obtained by the Northern blot approach, so the presence of one or both isoforms in lungs during inflammation and their concentrations were not specified. The fact that Cp is present in the protein fraction of normal human bronchial secretion indicates that extracellular Cp can be synthesized by the lungs [164]. However, its quantity does not exceed 13% of the serum. These findings are further supported by the presence of Cp synthesis and secretion in the culture of A549 cells [165]. It is not known if lung cells synthesize the GPI-Cp isoform. If GPI-Cp is present on the cell membrane of lung cells, it can hypothetically reduce IAV infectivity by competing with glycoprotein receptors for HA binding. At the same time, there is a probability that the presence of GPI-Cp on lung cell membranes may facilitate virus entry, as GPI-Cp is present in raft platforms, which are internalized by caveolin-mediated endocytosis. It is obvious that a thorough investigation of Cp gene expression in lung cells by modern methods is required to understand the mechanism of Cp-mediated antiviral protection.

The next question is the significance of Cp antioxidant properties for defense of the cells from ROS, produced in the course of the infection. However, in the extracellular space, Cu, Zn-SOD3 is present, which is comparable to cytosolic Cu, Zn-SOD1 in terms of catalytic activity. The Cp/SOD3 ratio in blood serum is about 2000:1 (0.3 mg/mL and 0.18 ug/mL, respectively). The ratio is significantly lower in lung extracellular fluid due to the low Cp concentration there, while in lung tissue, SOD3 is synthesized locally and lungs have the highest concentration of SOD3 among the organs [166]. Moreover, the superoxide dismutase activity of Cp is significantly lower than the SOD3 activity [167]. In total, all of these data suggest that the SOD activity of Cp is not important for protection against viral infection.

An unexpected role of Cp in the development of viral infection was discovered in experiments on the influence of silver nanoparticles (AgNPs) on IAV replication. It has been shown that AgNPs had a destructive effect on the virus membrane glycoprotein knobs. Likely due to this, the treatment of cells or viruses with AgNPs in nontoxic concentrations led to the blocking of virus entry to the cells and prevented apoptosis [168,169]. Recent discoveries that followed and supported these pioneering results were comprehensively analyzed in several reviews [170,171,172]. Moreover, intranasal AgNPs administration in mice enhanced survival after IAV infection. In AgNP-treated mice, the level of lung tissue damage was decreased, while weight loss was less profound [121]. It is interesting that intraperitoneal treatment of mice by a low dose of AgNPs (approximately 10 times lower than the minimum effective dose) caused a significant decrease in lethality during IAV infection, reduced the number of lesions in the lungs, and increased the mean survival time. At the same time, the virus titer in the lungs was not increased [120]. The largest antiviral effect was observed when mice were treated with AgNPs prior to infection and holo-Cp levels were reduced by 70% at the moment of inoculation.

*Point 6. More examples of the interference of copper metabolism and its effect on IAV reproduction*. In addition to the above examples of the link between influenza virus reproduction and copper ions, it can also be mentioned that copper ions are required for the formation of the virion [173]. Thus, IAV RNA-transcriptase activity is inhibited by copper chelators in vitro and in vivo [174]. Additionally, IAV, similar to SARS-CoV-2, causes loss of smell [175,176], and the co-activators of the odorant receptors are copper ions [62]. Moreover, IAV uses cell-signaling cascades for its reproduction, including copper-dependent signaling pathways [177,178]. Furthermore, one of the key stages of IAV reproduction (acidification of the endosome containing the virus) depends on M2, which is strongly and reversibly inhibited by copper ions. The wild-type M2 protein has very high specificity for copper(II), and the binding of copper results in an approximately equal inhibition of both inward and outward currents [179].

## 5. AgNPs as Potential Indirect Antivirus Agents

In mammals, the administration of AgNPs by intraperitoneal, oral, or intranasal means resulted in decreases in Cp oxidase activity [180,181]. This effect is caused by the replacement of copper in Cp active centers by silver ions. Ag(I) ions are isoelecronic to Cu(I) ions and have similar coordination properties, so they are recognized by copper transport proteins and transported to the lumen of the Golgi of liver cells [182,183,184]. The insertion of Ag(I) into Cp molecules results in the loss of the oxidase activity and prevents their normal folding. At the same time, Cp gene expression at the transcription and translation levels as well as glycosylation, secretion, and stability of the protein in the circulation are not affected by AgNPs. As a result, Cp oxidase activity in the blood serum of mice treated with AgNPs can be brought below the limit of detection while the normal concentration of the Cp protein in the blood is preserved [185].

It has been shown that holo-Cp derivatives that do not possess oxidase activity (apo-Cp or fragments of Cp molecule), contrary to holo-Cp, are not able to induce apoptosis in neutrophilsand release of cytokines. During IAV infection, both the quantity of neutrophils in the lungs and the local synthesis of holo-Cp increase. Thus, the danger of a cytokine storm, which is a severe condition responsible for lethality in the IAV infection, also increases. Therefore, a low level of holo-Cp may cause a decrease in the lethality of IAV-infected mice treated with AgNPs. It can be speculated that the effect of AgNPs saves mice from severe complications caused by IAV infection in the same way as the prion protein [117]. These results provide evidence that AgNPs may have beneficial effects on the course of IAV. AgNPs represent a good therapeutic agent against IAV infection, as the holo-Cp level is dampened by AgNP administration in only one day, and the effect is similar for different modes of administration. It is also beneficial that the holo-Cp level is rapidly recovered after treatment with AgNPs, and silver involved in their metabolism is excreted through bile [186]. AgNPs are also attractive because they induce resistance to flu at subtoxic concentrations [120,186].

## 6. Conclusions

Viruses’ acquired resistance to antiviral drugs is a substantial challenge in fields such as clinical practice and virology. From this perspective, the screening of compounds that are independent of the genetic variability of the target virus is of importance. In this review, we first attempted to analyze the data indicating that copper metabolism in both the host infected cell and the organism is used by IAV to implement its genetic program (Table 1). It cannot be ruled out that some of the conclusions made in the article will be further clarified or even refuted by more detailed studies.

The purpose of this review was to draw the attention of scientists to AgNPs as a tool for copper metabolism modulation. The reducing effect of AgNPs on Cp oxidase activity develops quickly and can also be quickly canceled. In terms of development time, this effect is comparable to influenza disease progression. The inhibitory effects of nanosilver on IAV in in vitro and in vivo experiments [120,121,168,169] allows for the conclusion that AgNPs can be a potential tool for the development of a preparation that efficiently eradicates influenza infection.

## Figures and Tables

**Table 1:** The potential relationships between copper metabolism and IAV replication.

Link between Copper Metabolism and IAV Replication	IAV Life Cycle Stage Sensitive to Copper Metabolism
Holo-ceruloplasmin	Holo-ceruloplasmin binds IAV. This can lead to a decrease in IAV infectivity.
Apo-ceruloplasmin	Does not stimulate neutrophil apoptosis. This may reduce the likelihood of developing a cytokine storm.
Prion protein	Copper transporter for CCS-independent SOD1 metallization and ROS concentration decrease.
Prion protein	Induction of the anti-inflammatory macrophage M2 maturation. This reduces the likelihood of developing a cytokine storm.
SOD1	Reduces the ROS concentration and suppresses the first stages of viral reproduction.
ATP7A-dependent autophagy	Activation of autophagy facilitates the release of the viral genome from endosomes and the production of infectious viral particles.
Copper import and excretion	Copper dyshomeostasis reduces IAV reproduction.
Binding with copper ions	Reversible inhibition of M2 protein, which is responsible for acidification of viral core proteins.
Binding with copper ions	Induction of IAV RNA-transcriptase activity.
